# Status and Opportunities of Machine Learning Applications in Obstructive Sleep Apnea: A Narrative Review

**DOI:** 10.1101/2025.02.27.25322950

**Published:** 2025-03-01

**Authors:** Matheus Lima Diniz Araujo, Trevor Winger, Samer Ghosn, Carl Saab, Jaideep Srivastava, Louis Kazaglis, Piyush Mathur, Reena Mehra

**Affiliations:** 1 Cleveland Clinic Foundation, Cleveland, OH, USA; 2 University of Minnesota, Minneapolis, MN, USA; 3 Epic Systems Corporation, Verona, WI, USA; 4 University of Washington, Seattle, WA, USA

**Keywords:** Sleep Apnea, Machine Learning

## Abstract

**Background:**

Obstructive sleep apnea (OSA) is a prevalent and potentially severe sleep disorder characterized by repeated interruptions in breathing during sleep. Machine learning models have been increasingly applied in various aspects of OSA research, including diagnosis, treatment optimization, and developing biomarkers for endotypes and disease mechanisms.

**Methods:**

This narrative review examines data extracted from 254 scientific publications published between 2018 and 2023 across a wide spectrum of research efforts, from diagnostic algorithms to treatment and patient management strategies. We evaluated the landscape of machine learning in OSA research by assessing the techniques used, application areas, model evaluation strategies, and dataset characteristics across studies.

**Results:**

Our analysis revealed that the majority of machine learning applications focused on OSA classification and diagnosis, utilizing various data sources such as polysomnography, electrocardiogram data, and wearable devices. Deep learning models were the most popular, followed by support vector machines, with classification tasks being the most common. We also found that study cohorts were predominantly overweight males, with an underrepresentation of women, younger obese adults, individuals over 60 years old, and diverse racial groups. Many studies had small sample sizes and limited use of robust model validation.

**Conclusion:**

Our findings highlight the need for more inclusive research approaches, starting with adequate data collection for better generalizability of machine learning models in OSA research. Addressing these demographic gaps and methodological opportunities is critical for ensuring more robust and equitable applications of artificial intelligence in healthcare.

## Introduction

Obstructive sleep apnea (OSA) affects a significant portion of the US population, with approximately 38% of the US population being diagnosed with mild severity and up to 17% diagnosed with moderate severity [1]. OSA is characterized by frequent, intermittent cessation (apnea) or reduction (hypopnea) of airflow during sleep, and it can severely affect individual health if left untreated, with serious negative ramifications for global health if unrecognized and untreated [2]. The gold standard for sleep apnea diagnosis is polysomnography (PSG), which records a large amount of patient data while sleeping, including brain waves, blood oxygen levels, heart rate, breathing, and eye and leg movements [3]. Additionally, Home Sleep Apnea Tests (HSATs) have emerged as practical diagnostic tools since patients can perform their sleep study at home. However, the HSAT approach is standardly less comprehensive than the in-laboratory PSG. A key diagnostic metric is the Apnea-Hypopnea Index (AHI), calculated based on the number of apneas and hypopneas events per hour of sleep derived from the sleep study. Although AHI tends to oversimplify the complex nature of individual OSA cases, clinicians widely use this metric, which traditionally considers 5, 15, and 30 events/hour thresholds to discriminate normal, mild, moderate, and severe cases, respectively [4], with diagnosis integrating the clinical presentation of the patient’s symptoms and comorbidities [5].

In sleep medicine, the study, diagnosis, prognosis, and treatment of OSA pose numerous challenges, from treatment adherence to diagnostic metrics. At the same time, OSA research provides uniquely rich data regarding demographic representation and high-density physiological signals that, when leveraged by innovative technologies such as applied machine learning and wearable devices, can address current OSA challenges and contribute to translational research across different medical domains [6].

Machine learning can extract complex patterns from data to perform classification and regression tasks, which is critical to reveal hidden knowledge in large volumes of data. For example, when seeking a new, low-cost, and feasible OSA diagnosis tool, [7] Wang used a deep learning algorithm based on sleep sounds for apnea detection. In another innovative task, [8] Araujo used a large cohort of thousands of patients receiving continuous positive airway pressure (CPAP) therapy for OSA management to train machine-learning models to predict when interventions are needed due to low adherence.

Several reviews have targeted machine learning applications in OSA. In one study, Mendonca et al. focused on analyzing algorithms for sleep apnea diagnoses based on pulse oximetry, electrocardiogram (ECG), and sound analyses [9]. In another study, Ramachandran et al. searched the literature for different types of sensors used for data collection and feature engineering techniques for sleep apnea detection [10]. Despite these reviews, to the best of our knowledge, no review has described and aggregated model training techniques, model performance, and their dataset characteristics.

Here, we compare specific details of machine learning approaches, such as model type, evaluation metrics employed, and the detailed characteristics of the datasets, including individual-level demographics. In this review, we conduct a comprehensive overview of the current status of machine learning in OSA research. Our objectives for this review are to re-evaluate current data modeling methodologies, understand potential ethical biases, and chart the way forward using state-of-the-art artificial intelligence in healthcare research.

## Methods

We conducted our review by initially identifying search terms that align with our purpose and research questions, employing an iterative approach to select studies and manually extract data. We then performed a numerical summary of key data elements, followed by a report on the results and a discussion of the findings with specific attention to their implications for clinical practice, research, and future investigations into machine learning applications in OSA.

Our literature review was conducted using PubMed (National Library of Medicine, Bethesda, MD) advanced search to identify studies released from January 2018 to March 2023. PubMed was chosen because it is a comprehensive and authoritative database widely used in biomedical research, ensuring access to high-quality, peer-reviewed articles relevant to sleep apnea and machine learning. Non-human studies were excluded.

We searched for machine learning-related terms associated with OSA for the search criteria, resulting in following search query: *(((‘Machine*-*learning’) OR (‘Artificial*-*intelligence’) OR (‘Neural*-*networks’) OR (‘Deep*-*learning’) OR (‘Reinforcement*-*learning’) OR (‘Predictive*-*modeling’) OR (‘Statistical*-*learning’) OR (‘Computer*-*vision’) OR (‘Natural*-*language*-*processing’) OR (‘Decision*-*trees’) OR (‘Random*-*forests’) OR (‘Support*-*vector*-*machines’) OR (‘Clustering’) OR (‘Convolutional*-*neural*-*networks’) OR (‘Recurrent*-*neural*-*networks’) OR (‘Generative*-*adversarial*-*networks’) OR (‘Transfer*-*learning’) OR (‘Ensemble*-*learning’) OR (‘Unsupervised*-*learning’) OR (‘Supervised*-*learning’) OR (‘Semi*-*supervised*-*learning’) OR (‘Active*-*learning’) OR (‘Bayesian*-*networks’) OR (‘Decision*-*tree’) OR (‘Random*-*forest’) OR (‘Support*-*vector*-*machines’) OR (‘SVM’) OR (‘Naive*-*Bayes’) OR (‘K*-*nearest*-*neighbors’) OR (‘KNN’) OR (‘Principal*-*component*-*analysis’) OR (‘PCA’) OR (‘Recurrent*-*neural*-*networks’) OR (‘RNN’) OR (‘Convolutional*-*neural*-*networks’) OR (‘CNN’) OR (‘Generative*-*adversarial*-*networks’) OR (‘GANs’) OR (‘Autoencoders’) OR (‘Hidden*-*Markov*-*models’) OR (‘HMMs’) OR (‘Long*-*short*-*term*-*memory’) OR (‘LSTM’)) AND ((‘obstructive*-*sleep*-*apnea’)*.

**Figure 1:**
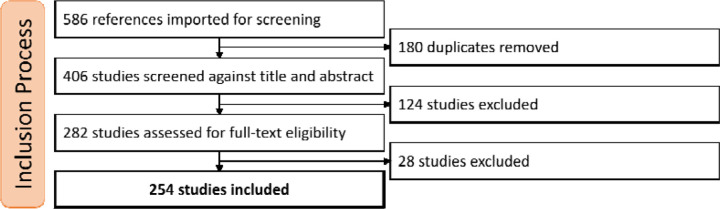
Inclusion process diagram. Diagram showing the survey including process with 3 major filtering steps.

Our query returned 586 references using the search criteria. These references underwent three filtering processes, as shown in Figure 1. First, we removed 180 duplicate references. Then, we excluded 124 studies that were not relevant based on title and abstract analysis. Finally, we removed 27 studies after analyzing the complete text because they were not original publications, did not use machine learning in their methodology, were non-English publications, or were related to other non-obstructive sleep apneas. The final dataset for data extraction included 254 studies. For the screening and data extraction process, we used Covidence^1^ software (Veritas Health Innovation, Brooklyn, NY). The full reference of each included study is available in the supplemental materials.

We performed manual data extraction from the full text of each study with subsequent second-person revision. While screening the text, our group extracted the main application of machine learning from each article, categorizing them into 18 major subjects. We extracted the following machine learning-related variables: the type of model used (e.g., logistic regression, random forest), task type (e.g., classification, regression), and traditional validation metrics (e.g., accuracy, precision, recall, area under the ROC curve). We also extracted information about the data source (e.g., polysomnogram, questionnaires, electronic health records) and the data type (e.g., tabular data, raw audio). Moreover, demographic sample information was also extracted (e.g., age, sex, race, BMI). We removed studies that did not have the reported information for each analysis. This data extraction and categorization process enabled our quantitative thematic analysis.

## Results

### Analysis of Studies Subject Categories

Table 1 presents the distribution of 18 machine-learning application topics in OSA research. The primary focus of the studies, representing 30% (77 studies), was on using machine learning to classify sleep apnea diagnosis. Other significant categories include the automatic identification of hypopnea/apnea events at the epoch level (11%) and the detection and profiling of snoring (10%). Less common applications involve the severity stratification of sleep apnea using the Apnea-Hypopnea Index (AHI) (8%), the prediction of mortality and cardiovascular risks (7%), and the automatic classification of sleep stages (7%), reflecting the diverse potential of machine learning in enhancing diagnostic and predictive accuracy in sleep medicine. The table shows the broad spectrum of research, including categories focusing on novel technologies (e.g., wearable devices) for diagnosis, machine learning in genetic screening, and treatment-related applications (e.g., CPAP adherence) [11–13].

### Demographic analysis of age, sex, body mass index, and race

We recorded several key demographic characteristics that provided insight into the participant profiles across various studies, including age, sex, BMI, and race.

**Figure 2:**
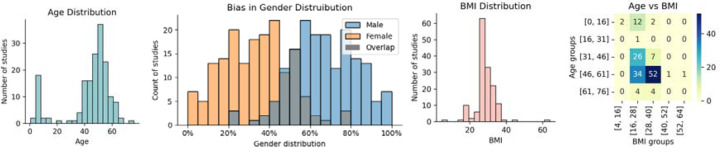
Mean demographic data distribution. (a) Mean age distribution, (b) gender distribution across publications highlighting gender bias, (c) mean BMI distribution and (d) heatmap showing the number of studies by BMI and age groups.

The age distribution of the study population reveals a distinct bimodal pattern, as shown in Figure 2a. This pattern highlights a primary focus on pediatric research and individuals who are 40–60 years old.

The participant’s BMI across various studies was 28.2±6.18kg/m^2^, indicating a prevalent trend toward the overweight but not severely obese population. The presence of extreme values, as seen in Figure 2c, suggests variability in the health profiles of the participants, which may include the pediatric population with relatively low BMI. The heatmap of both mean age and BMI groups in Figure 2d indicates potential gaps in research, such as a low representation of studies with younger obese adults and low representation of older populations (i.e., populations with a higher prevalence of OSA).

Sex-specific distributions varied significantly across studies, as shown by the sex distribution histograms in Figure 2b. On average, males constituted 67±16% of the samples, in contrast with females, who constituted an average of 33±16%, which is consistent with the higher prevalence of OSA in men versus women.

The studies span multi-national geographical locations, including countries across Asia, Europe, and America. Race or ethnicity is mentioned in 142 studies (55%), but only 20 (8%) explicitly provided a multiracial or multiethnic sample participation description.

### Analysis of Data Source and Types

**Figure 3:**
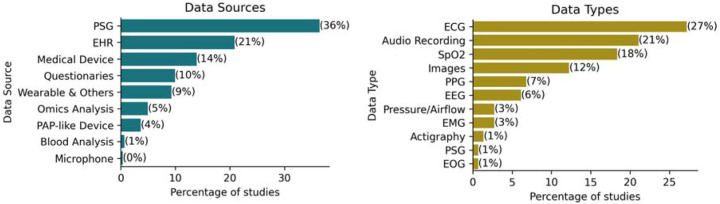
Popularity of Data Types (left) and Data Sources (right).

We distinguished between the sources of data and their respective types. The source indicates the high-level origin of data, and they included *-omics analysis, polysomnography (PSG), medical devices, electronic health records (EHR), questionnaires, wearables and other devices, positive airway pressure (PAP)-devices, blood analysis, and microphones. Each source may encompass multiple data types. For instance, in a polysomnogram, data collected include electrocardiogram (ECG), oxygen saturation (SpO2), and electroencephalogram (EEG) types. The distribution of each data source and type across the studies is detailed in Figure 3.

We found that 83 studies (32%) utilized multiple data sources, underscoring the multimodal nature of data integration in OSA research. The most used data type was ECG, which appeared in 27% of the studies, which was mostly derived from PSG (36%). Following PSG, EHR was utilized in 21% of the studies. Interestingly, despite representing the primary treatment for OSA, data from Positive Airway Pressure (PAP) devices were available in only 4% of the studies.

### Choice of machine learning algorithms over time

**Figure 4:**
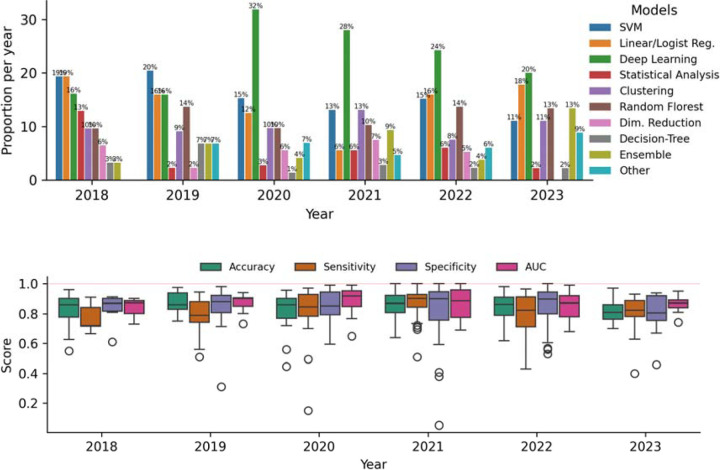
(Top) Popularity of machine learning models per year. (Bottom) Aggregate performance metrics over time

We assessed the popularity of machine learning models over the years(Figure 4 - Top). Deep Learning models are the most popular, followed by support vector machines, which have a negative trend, and Ensemble methods like XGBoost and Random Forest models, which have an increasing trend. Traditional linear models have remained consistent in popularity over the years.

### Analysis of Classification Evaluation Metrics

We evaluated the types of machine learning tasks addressed in each study, focusing on classification, clustering, and regression. Classification was the most common task, appearing in 195 studies. Given this prevalence, we conducted a detailed analysis of classification-related performance metrics. The most frequently reported metrics were accuracy (163 studies), sensitivity (127 studies), specificity (123 studies), and area under the receiver operating characteristic curve (AUC, 106 studies). Figure 4 presents the distribution of these metric scores over the years, which typically ranged from 0.78 to 0.92, with an average of 0.83. Papers published in 2018 showed comparatively lower scores than those in subsequent years. Additionally, some papers from 2020, 2021, and 2022 reported perfect scores of 100%.

### Sample Size, Test Size, and Validation Strategy

**Figure 5:**
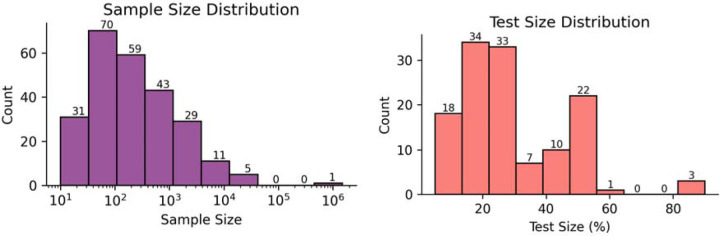
Dataset size and test-set representation. (a) Distribution of Sample Size (log scale) and (b) the proportion of data separated for test.

Our analysis indicates that a wide range of sample sizes are used as datasets for machine learning applied to OSA. In Figure 5 (a), we show the logarithmic distribution of the sample size, where we can highlight that most studies (77%) have sample sizes up to 1000 samples. Only 6 studies utilized a sample size of more than 10000 samples, and only one study used more than 1 million samples, which used a large sample to determine the most critical predictor of time to death between central and obstructive sleep apnea groups [14]. Additionally, Figure 5(b) shows the proportion of data allocated for testing the machine learning models. We found that the majority of studies reserved up to 30% of their data for testing. The most common test size was 20%, used in 28 studies, followed by 30% in 26 studies and 50% in 18 studies.

Our findings reveal that some studies employ robust validation methods, such as 10-fold cross-validation in 52 studies (20%) and 5-fold cross-validation used in 33 studies (12%). Also, 28 studies (11%) reported using the leave-one-out cross-validation technique; this occurred most frequently in smaller datasets or studies where maximum utilization of available data was critical.

## Discussion

The main conclusions from this narrative review of existing published data focused on machine learning applications in OSA are as follows: 1) the majority of machine learning has focused on OSA classification and categorization; 2) cohorts studied were overweight, mostly men and although there was multi-national representation, were of unknown race distribution, 3) multiple data sources have been used to implement AI approaches with the most common being ECG, 4) the most common machine learning models used were deep learning followed by support vector machines (SVMs), 5) classification was the most commonly used evaluation metric and 6) a wide range of sample sizes was used with very few above 1000 and only half using validation approaches.

Our findings highlight the diverse applications of machine learning OSA research, with the primary focus on improving diagnostic methods. This trend is likely driven by the high costs of complex PSG procedures, which require patients to stay overnight in sleep laboratories. The emergence of innovative sensors in wearable devices, along with advancements in machine learning techniques, further underscores the interest in developing more efficient diagnostic methodologies [15]. An interesting example is the work of Gu et al. (2020), who used a novel pulse oximeter combined with an accelerometer and a trained neural network to compute the Apnea-Hypopnea Index (AHI), achieving a Pearson correlation score of 0.9 [16]. This study demonstrates the potential of wearables and machine learning in OSA diagnosis. However, we note a significant gap in the application of machine learning for OSA treatment. Although some studies on wearables address treatment, they account for less than 10% of the total studies, highlighting an opportunity to leverage trained models for long-term therapy management.

In our demographic analysis, we were interested in the different representations of these characteristics in current state-of-the-art research, thus shedding light on the heterogeneity of the current research landscape. Considering age and BMI, most of the populations in the studies were overweight but not severely obese. The age distribution shows a bimodal pattern, capturing both pediatric participants and adults in the 40–60-year age range, which are critical periods for the emergence of OSA risk factors and symptoms [17]. We identified significant gaps in research, specifically the low representation of studies involving younger obese adults and populations older than 60 years. The growing number of older individuals in most developed countries, coupled with the higher prevalence of OSA in these groups, highlights a critical gap in the literature. Our sex-specific analysis also revealed a higher prevalence of males versus females, which, although approximates OSA distribution in the general population, underscores the importance of considering enriching samples with women to better understand sex as a biological variable in clinical research. Geographically, the studies are well-distributed across continents. Still, the lack of data on reported race and ethnicity reveals a potential gap in inclusivity, with a small number of studies explicitly including multiracial or multiethnic samples. This demographic overview highlights the need for more balanced and inclusive research approaches to better address OSA across different populations. Moreover, this finding hits the current core demand for a discussion on machine learning ethics and how careful consideration is needed in terms of ethical guidelines to ensure equitable and unbiased models [18].

We found that 83 studies (32%) utilized multiple data sources, underscoring the complexity of data integration in OSA research where a multi-source of physiological data highlights the need for a multi-modal approach in machine learning. Consequently, we highlight the need to standardize data collection and processing methods to ensure consistency and comparability across studies and enhance the possibility of using machine learning models across different sites. We also observed that many studies leveraged open datasets, such as Physionet’s Apnea ECG dataset (Penzel et al., 2000), matching with the most frequent data type in our studies, at 27%. This highlights the crucial role of open databases in driving research, as without these resources, it is likely that many of the studies would not have been possible.

Given the complex nature of most of the datasets, we anticipated that deep learning models would be popular due to their capability to handle multi-modal and raw data. Additionally, the frequent use of tabular data that includes demographics and questionnaires is aligned with the high popularity of SVM and ensemble methods, which are current state-of-the-art techniques for such data. A notable observation was the small sample sizes in many studies, with more than half involving fewer than 1,000 samples. While this sample size is considered low in the broader machine learning field, it is understandable given the challenges of acquiring raw data in healthcare and the low availability of open datasets [19]. The distribution of test sizes was also as expected, adhering to the common practice of reserving approximately 20% of data for testing and the standard 10 or 5 folds in cross-validation.

The high frequency of each type of machine learning model analyzed was anticipated since deep learning is a very hot research topic and appropriate for multi-modal raw data. Also, the use of tabular data, including demographics and questionnaires, SVM models, and Ensemble methods, are the current state-of-the-art techniques for this type of data. Surprisingly, the sample size is smaller than expected with studies with less than 1000 samples. In the general machine learning field, such a number is considered low but understandable due to the difficulty of acquiring raw data in health care. The test size distribution was also expected since it reflects the current rule of thumb regarding the percentage of data separate for testing (20%) and the number of folds in cross-validation.

In terms of evaluation metrics for classification, we observed that most studies reported values around 80% independent of the metric, with a few studies achieving 100% for certain metrics. Perfect scores are extremely rare, especially in large datasets, which raises two significant concerns: 1) the possibility of inflated scores and 2) the risk of overfitting machine learning models. In the case of overfitting, the model’s performance is not reproducible outside their own research. The case of inflated performance requires community collaboration towards better reproducible research in terms of methodology (including research source code) and data availability, which are rare.

The limitations of this study include the temporal cutoff of March 2023, which means more recent advancements in the machine learning field and studies of large language models (LLMs) in obstructive sleep apnea (OSA) research were not considered. We also highlighted the limited number of analyses, where more research opportunities exist, especially in the analysis of potential machine learning bias due to biased demographic stratification.

Based on our narrative review, we propose several future research directions. A more detailed analysis of scientific studies on the scores and evaluation metrics used per machine learning technique and within each application category is needed. Ethical bias should be a part of every investigation involving model training to identify potential biases beyond those highlighted in this work. Additionally, exploring other sleep disorders, such as central sleep apnea and hypersomnia, may reveal other disorder-specific data biases. Future reviews should also include novel deep-learning approaches, including generative artificial intelligence, to uncover methodological limitations yet to be discovered.

## Conclusion

We reviewed 254 scientific articles that use machine learning in obstructive sleep apnea research, covering quantitative analysis of demographics of study samples, the range of tasks addressed by different machine learning techniques, and the validation strategies used. While we found that trained models are more often used to improve the diagnosis costs, there is an underrepresentation of women, younger obese adults, individuals over 60 years old, and diverse racial groups. Additionally, we found a prevalence of small sample sizes for training machine learning models, highlighting the challenges in healthcare data acquisition. Addressing these demographic gaps and standardizing data collection and processing methods are critical for advancing the field and ensuring more robust OSA research.

## Figures and Tables

**Table 1:** Absolute and relative popularity of studies categories. One study can have more than one study category.

Machine Learning Applications	Number of studies	Proportion (N=254)
Sleep apnea diagnosis classification	74	29%
Identification of hypopnea/apnea at epoch level	29	11%
Snoring detection and profiling	25	10%
Severity stratification (AHI)	20	8%
Predict mortality risk, cardiovascular risk, and other conditions related to Sleep Apnea	19	7%
Automatic classification of sleep stage in sleep apnea	19	7%
Detecting or screening other conditions related to Sleep apnea (e.g adenoid hypertrophy)	16	6%
Wearable or nearable devices	15	6%
Screening and profiling of protein/DNA/genetic/blood analysis for sleep apnea	14	5%
Clustering analysis for phenotyping those with sleep apnea	13	5%
OSA questionnaires analysis	9	4%
CPAP adherence and usage	8	3%
Electronic Health Records (EHR) analysis	7	3%
Sleep posture classification	5	2%
Predicting treatment outcomes	3	1%
Predicting candidate for surgery	3	1%
Predicting score results for drug-induced sleep endoscopy	3	1%
Obstructive Sleep Apnea vs Central Sleep Apnea	3	1%

## Summary Table

- The review analyzed 254 studies published between 2018 and 2023, finding that most applied machine learning to classify and diagnose obstructive sleep apnea (OSA), with deep learning models being the most popular techniques used.
- There are severe demographic gaps where most study populations were predominantly overweight males and an underrepresentation of women, younger obese adults, individuals over 60 years old, and diverse racial groups, indicating a need for more inclusive research to improve generalizability.
- Multiple data sources, such as polysomnography, wearables, and electrocardiograms, were used, but data collection methods lacked standardization. Many studies had small sample sizes and limited use of robust machine learning validation strategies, potentially affecting the reliability of machine learning applications.
